# Cardiovascular Risks of Simultaneous Use of Alcohol and Cocaine—A Systematic Review

**DOI:** 10.3390/jcm13051475

**Published:** 2024-03-04

**Authors:** Jan van Amsterdam, Femke Gresnigt, Wim van den Brink

**Affiliations:** 1 Department of Psychiatry, Amsterdam UMC, University of Amsterdam, Meibergdreef 9, 1012 WP Amsterdam, The Netherlands; jan.van.amsterdam@amsterdamumc.nl; 2 Amsterdam Neuroscience, Research Program Compulsivity, Impulsivity & Attention, P.O. Box 22660, 1100 DD Amsterdam, The Netherlands; 3 Emergency Department, OLVG Hospital, Oosterpark 9, 1091 AC Amsterdam, The Netherlands; f.m.j.gresnigt@olvg.nl; 4 Dutch Poison Information Center, UMC Utrecht, University Utrecht, 3508 GA Utrecht, The Netherlands

**Keywords:** cocaine, alcohol, cocaethylene, myocardial infarction, angina pectoris, mortality, emergency department

## Abstract

**Background**: The simultaneous use of cocaine and alcohol is highly prevalent and is associated with high numbers of emergency department admissions, primarily due to cardiovascular complications. Aims: To answer the question of whether the co-use of cocaine and alcohol increases the cardiovascular risk compared to the use of cocaine alone. **Method**: A systematic review of human studies comparing the cardiovascular risk of co-used cocaine and alcohol with the use of cocaine alone. **Results**: Despite a higher myocardial workload induced by the co-use of cocaine and alcohol and the potentiation of cocaine’s cardiovascular effects by alcohol, the findings on the risk and severity of cardiovascular symptoms due to combined use are inconsistent. However, the co-use of cocaine and alcohol clearly leads to higher mortality. Interestingly, the presence of cocaethylene, a unique metabolite generated only via a pharmacokinetic interaction between alcohol and cocaine, carries an 18- to 25-fold increase over the absence of cocaethylene (cocaine-alone users) in the risk of sudden death and is associated with myocardial injury and cardiac arrest, probably due to the inhibition of cardiac ion channels by cocaethylene. **Conclusion**: Despite the inconsistency in some of the results, it is concluded that the co-use of cocaine and alcohol poses an additional risk of cardiovascular fatalities compared to the use of cocaine alone.

## 1. Introduction

Cocaine is frequently co-used with alcohol. It is estimated that some 92% of cocaine users drink alcohol [1] and a meta-analysis of ten studies (1985–2017), mainly from community samples from the U.S. and Canada, estimated that 74% (range 24–98%) of all cocaine users also simultaneously use alcohol [2]. Similarly, the prevalence of alcohol use among cocaine-dependent subjects was 89% [3].

As a potent sympathomimetic drug, cocaine is associated with considerable cardiovascular complications [4,5,6,7,8,9,10,11], including angina pectoris, coronary vasospasm, myocardial ischemia, acute myocardial infarction (AMI), cerebrovascular disease, ventricular arrhythmias, and sudden cardiac death. Therefore, it is no surprise that cocaine is one of the main causes of drug-related emergency department (ED) visits, due to cardiovascular problems [11], with chest pain mentioned as the most common complaint [12,13]. Of all patients attending an ED with non-traumatic chest pain, 14–25% in urban hospitals and 7% in suburban hospitals have detectable levels of cocaine or cocaine metabolites in their urine [14]. The latter is worrying because some 4–6% of patients with cocaine-associated chest pain in the ED developed AMI [15,16,17]. Furthermore, in the first hours after cocaine use, the risk for AMI in ED patients was 4.3 to 24 times higher than in non-cocaine users [17,18], and a history of cocaine use was associated with an almost six times higher risk for an acute coronary syndrome [19].

Importantly, in 2011, the Drug Abuse Warning Network (DAWN), a voluntary ED data collection system on medical crises and deaths related to the use of drugs in the U.S., reported that cocaine was the most common substance resulting in ED attendance (*n* = 505,224 visits; 40.3% of all reported drug-related visits). The co-use of cocaine and alcohol was the number one drug combination leading to 173,799 ED visits (29% of cocaine-related ED visits) [20], which is still relatively low considering the high popularity of combined cocaine and alcohol use. More recently, due to the emerging use of (synthetic) opioids and methamphetamine, the proportion of cocaine-related ED visits reported by the DAWN declined to only 4.7% of all drug-related ED visits in 2021 [21]. In Europe, cocaine use was still responsible for 21% of all drug-related ED presentations in 2020 (cannabis: 23%), but no data were reported for the simultaneous use of cocaine and alcohol (further denoted as cocaine–alcohol) [22]. A recent Spanish ED study performed between 2017 and 2019 reported that the co-ingestion of alcohol with either cocaine or cannabis was the most prevalent drug combination seen in ED admissions with 32.2% and 24.0% of all drug-related ED visits, respectively [23]. In Sweden, 60% of the 2627 cocaine-positive samples from acute drug-related poisonings (2010–2016) were also positive for alcohol [24]. Similarly, 72% of cocaine users who attended an ED in Colombia between 2016 and 2017 also tested positive for alcohol [25]. In the U.S., 19% of all cocaine-related ED visits also had a co-occurring alcohol-related diagnosis during the same ED visit (19%; 95% CI: 15–25%) [26].

However, it remains to be resolved whether the simultaneous use of cocaine and alcohol leads to more severe complications compared to the use of cocaine alone, which may lead to a higher number of ED admissions. The current systematic review therefore outlines the cardiovascular complications of alcohol combined with cocaine compared to those following the use of cocaine alone, including the underlying interactions between both substances.

## 2. Methods

**Figure 1:**
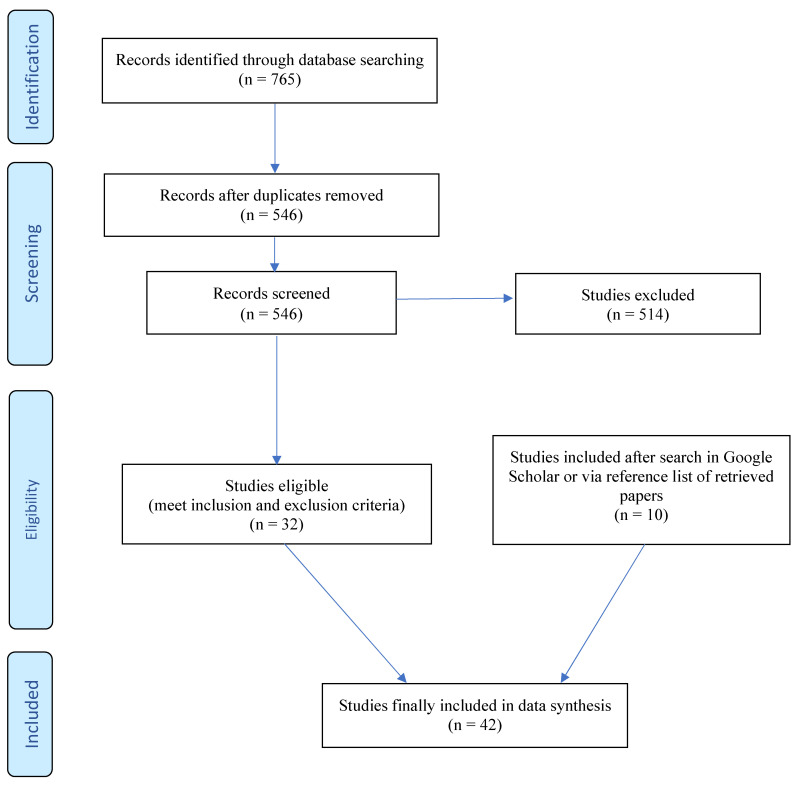
PRISMA flow diagram. Additional eligible reports were retrieved by checking the reference lists of the selected 42 studies.

Using the PRISMA protocol, a systematic literature review (registration number: CRD42024002405) was performed on 30 November 2023 to retrieve studies from Medline (PubMed) and Embase on acute adverse cardiovascular effects after the concurrent use of alcohol and cocaine. Inclusion criteria: studies were required to be conducted in human participants comparing the co-use of alcohol and cocaine (i.e., cocaine–alcohol) with either cocaine alone, alcohol alone, or non-use of either. The selection of eligible studies was independently performed by JvA and WvdB in two rounds. A total of 765 studies were identified from the initial search and 546 articles remained after duplicates were removed. These 546 studies were further processed, i.e., the title and abstract were screened to determine eligibility by applying the above inclusion and exclusion criteria. In a second round, the full texts of the selected 32 studies were screened for eligibility. In addition, ten additional relevant studies were retrieved via the reference lists of the retrieved papers or via Google (Scholar) using appropriate keywords, resulting in 42 eligible studies being included. Figure 1 shows the PRISMA flow chart for the identification, screening, and inclusion of the studies. See ‘Supplementary Materials’ for the search string and PRISMA checklist.

## 3. Results

### 3.1. Pharmacodynamic Effects of Cocaine

**Figure 2:**
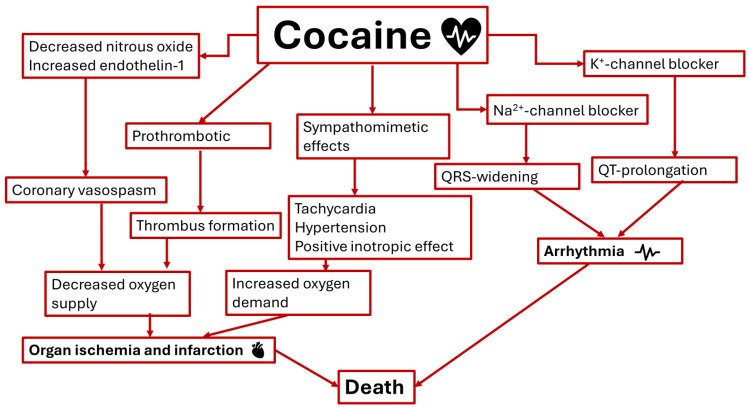
Simplified schematic presentation of the cardiotoxicity of cocaine (F.M.J. Gresnigt©).

Cocaine is a sympathomimetic drug that inhibits the reuptake and increases receptor sensitivity for (nor)adrenaline, dopamine, and serotonin. As reviewed by Kim and Park (2019) [4] and depicted in Figure 2, cocaine’s cardiotoxicity is caused by multiple mechanisms. Firstly, increased sympathomimetic stimulation results in increased heart rate (HR), blood pressure (BP), and myocardial contractility, leading to increased myocardial oxygen demand. Secondly, the noradrenaline effect causes vasoconstriction and, combined with the prothrombotic effect, decreases the oxygen supply. Therefore, the increased myocardial oxygen demand may exceed the myocardial oxygen supply, which may in turn lead to myocardial ischemia or infarction. Thus, cocaine can cause AMI, which is the most frequent severe cardiovascular complication in cocaine users [18,27]. Finally, cocaine directly affects the sodium and potassium channels, impairing the transmission of the nervous impulses of the heart. The mechanisms of cardiotoxicity will be further explained in detail below.

### 3.2. Pharmacodynamic Effects of Alcohol

Alcohol alone has a mild acute direct effect on hemodynamics. In healthy human subjects, moderate doses of alcohol cause a transient and slight increase in HR, systolic blood pressure (SBP), diastolic blood pressure (DBP), and cardiac output [28]. Alcohol levels below blood concentrations of 190 mg/dL (2‰) do not affect coronary blood flow [29]. A recent Cochrane review further showed that a low to moderate dose of alcohol had minor acute effects on HR and BP. Within six hours of consumption, low-dose alcohol (<14 g) did not affect the BP but did increase the HR by 5.1 bpm, while medium-dose alcohol (14 to 28 g) decreased the SBP and DBP by 5.6 mmHg and 4.0 mmHg, respectively, and increased the HR by 4.6 bpm [30]. In the 24 h after alcohol consumption, the risk of fatal and non-fatal coronary heart disease [31], including AMI [32], was reduced. Interestingly, within one hour after alcohol consumption, the incidence rate of AMI was 1.7-fold higher among people who do not typically drink alcohol daily but not among daily drinkers [33].

### 3.3. Pharmacokinetic Interactions of Cocaine and Alcohol in Humans

As outlined below, alcohol may aggravate cocaine’s cardiotoxicity via a pharmacokinetic interaction, resulting in (a) increased cocaine plasma levels and (b) the formation of the cardiotoxic metabolite cocaethylene (CE).

(a) Increased cocaine plasma levels

In humans, cocaine is hydrolyzed to benzoylecgonine and ecgonine methyl ester or demethylated to norcocaine. In experienced but non-dependent cocaine users, the simultaneous oral use of alcohol and cocaine results in higher cocaine plasma levels, possibly due to a faster rate of cocaine absorption or as a result of the inhibition of hepatic cocaine metabolism by alcohol, i.e., alcohol suppresses the first-pass metabolism of cocaine in the liver [34,35,36,37,38,39]. This enhancing effect of alcohol on cocaine plasma levels depends, however, on the timing and order of consumption of the two substances and is only relevant when cocaine is used orally [40,41]. Alcohol does not increase the cocaine plasma level when cocaine is used via the more common routes, i.e., intravenously or through smoking, because these routes bypass the first-pass metabolism of cocaine, though alcohol has a small effect when cocaine is used intranasally [34,41,42,43]. Generally, cocaine peak plasma levels and the AUC (area under the curve; total cocaine exposure across time) were higher following cocaine–alcohol than in the cocaine-alone condition, and cocaine plasma clearance was reduced by 50% without changes in the elimination half-life [42]. Compared to intranasal cocaine alone, its simultaneous use with alcohol (1 g/kg) led to 18–20% higher cocaine peak plasma levels and a 23–29% higher AUC_0–360_ in humans [34,42], whereas pre-treatment with alcohol (1 g/kg p.o.) did not affect the bioavailability of cocaine when cocaine was administered intravenously [43].

(b) Formation of the cardiotoxic metabolite cocaethylene (CE)

CE is an active cocaine metabolite only formed in the liver when alcohol and cocaine are used within approximately 2 h of each other. CE can, therefore, be used as a specific biomarker for the simultaneous consumption of cocaine and alcohol. The proportion of cocaine converted to CE depends on cocaine’s route of administration: 34% (oral), 17–24% (i.v.), and 18% (smoking) [44,45]. In contrast to benzoylecgonine, the major inactive metabolite of cocaine, CE, is not only active but also has a plasma elimination half-life of about 2 h, which is 2–5 times that of cocaine [38,41,46,47]. However, only (very) little CE is produced at low cocaine plasma levels, regardless of the amount of alcohol consumed [40].

In summary, the pharmacokinetic interaction between alcohol and cocaine, when simultaneously used, increases cocaine peak plasma levels by 18–20% and the cocaine plasma AUC by 23–29% compared with cocaine alone. In addition, cocaine–alcohol results in the formation of CE, an active cocaine metabolite with a plasma elimination half-life of 2–5 times that of cocaine, making its effects more persistent.

### 3.4. Pharmacodynamic Interactions of Cocaine and Alcohol

In humans, cocaine alone (2 mg/kg, intranasal) increased the HR (17 ± 16% bpm), the mean arterial pressure (MAP: ±7% mm Hg), the cardiac index (18 ± 18% L/min/m^2^), and ventricular contractility (dP/dt: 18 ± 20% mm Hg/s) [48]. Cocaine–alcohol produced a significantly higher increase in the HR (plus 16–20%) and a variable, but generally small, increase in the SBP or DBP when compared with either the placebo, cocaine alone, or alcohol alone [36,37,38,42,49,50]. In an earlier study, performed in nine healthy male volunteers, cocaine–alcohol increased the HR by up to 40 bpm [35] compared to a significant increase in the HR by about 6 bpm after cocaine alone (48 and 96 mg inhaled) and an increased HR by 4 bpm after alcohol alone (19 to 58 g p.o.). The product of the HR and the SBP, termed the rate pressure product, is a very reliable indicator of myocardial oxygen demand. Thus, cocaine–alcohol induces an additional increase in myocardial oxygen consumption (cardiac output; HR x stroke volume) compared to cocaine alone. Indeed, patients with chest pain, who were referred for catheterization and were given intranasal cocaine (2 mg/kg) plus alcohol (5 mL/kg), showed an increased myocardial oxygen demand of 17% and 16% at 30 and 90 min, respectively, with a concomitant increase of the coronary arterial diameter by 7% and 13%, respectively, whereas in patients who were given cocaine only, the myocardial oxygen demand increased by 5% and 10%, respectively. Surprisingly, and in contrast to previous findings showing coronary vasoconstriction by intranasal cocaine [51,52], the coronary artery diameter increased by 14% at the same time points [53]. This is of interest because coronary artery vasoconstriction would further impair the oxygen supply to the heart [51].

Alcohol may also potentiate the actions of cocaine via the formation of CE [50]. CE has a pharmacological profile similar to that of cocaine [38] and also induces cardiovascular effects, like tachycardia, comparable or smaller than those observed after cocaine alone [54,55,56,57,58,59]. For instance, CE (0.25 mg/kg, i.v.) produced a 43% lower elevation of the HR and BP than the same dose of cocaine alone [56,59] or comparable tachycardic effects as cocaine alone [55,58]. These smaller effects of CE compared with cocaine alone are not in agreement with those described above, showing a larger effect of intranasal cocaine–alcohol on the HR compared to cocaine alone [35]. A comparison of (non-traumatic) patients attending an ED who screened positive for benzoylecgonine (*n* = 149; cocaine-alone users) with those positive for benzoylecgonine and alcohol (*n* = 63; cocaine–alcohol) showed that co-users presented with a higher HR and BP on admission than cocaine-alone patients.

In summary, the significant tachycardia (plus 16–20%) and the minimal (inconsistent) increase in BP following the administration of intranasal cocaine–alcohol implicates a higher myocardial oxygen demand, which may explain the higher cardiotoxicity seen in co-users compared to those using cocaine alone. CE showed cardiovascular effects grossly similar to cocaine alone, leaving it unclear whether CE really poses an additional pharmacodynamic cardiovascular risk.

### 3.5. Cocaine and Alcohol Interactions in Cardiac Arrhythmia

Cocaine inhibits, as illustrated in Figure 2, electrical nerve transmission via the blockade of fast inward sodium channels and the inhibition of the hERG/IKr (*human ether-a-go-go-related* gene; HERG) channels serving the outward current of potassium [60]. The tachycardia induced by cocaine further boosts the degree of sodium channel blockade [61]. As such, the upstroke of the action potential (phase 0) is delayed with resultant prolongation of depolarization, a prolonged QRS-interval, impaired myocardial contractility, and cardiac arrhythmia [6,62], and the repolarization (phase 3) is prolonged, resulting in a prolonged QT interval [63]. QT-prolongation is a risk factor for ventricular arrhythmias such as ventricular tachycardia and Torsade de Pointes and may result in sudden cardiac death. Fatal cardiac arrhythmias are often suspected in sudden cocaine-related deaths [64]. Compared to cocaine, CE is an even more potent blocker of cardiac sodium channels (K_d,i_ of 7.9 μM and 5.1 μM, respectively) [65] and a 3-fold more potent blocker of HERG potassium channels (IC_50_ of 4.4 μM and 1.2 μM, respectively) [60], which could explain its higher cardiotoxicity, in particular, cardiac arrhythmia. The role of acute alcohol consumption in arrhythmia highly depends on the dose and these effects are not well understood. Excessive alcohol intake may lead to sudden cardiac death, whereas moderate alcohol intake may have anti-arrhythmic properties [66,67].

Recent cocaine use among young people was associated with an up to 4-fold increased risk of cardiac arrest [68], whereas—except for one short poster abstract—no data could be retrieved about cocaine–alcohol-related sudden death. This abstract [69] described subjects attending the ED following cocaine–alcohol use (*n* = 172) and showed significantly larger QTc-dispersion (95% CI: 58.4–82.1 ms) compared to users of cocaine alone (95% CI: 24.3–42.9 ms) or alcohol alone (95% CI: 34.0–45.5 ms; *p* < 0.001). P-wave dispersion, a predictor for atrial fibrillation, was also significantly greater in patients who used cocaine–alcohol (95% CI: 50.9–68.2 ms) compared to cocaine alone (95% CI: 31.7–44.4 ms; *p* < 0.001) [69].

In summary, due to its blockade of cardiac sodium and potassium channels, cocaine use may elicit cardiac arrhythmia, which may have a fatal outcome. Compared to cocaine alone, CE is a more potent blocker of both cardiac sodium and potassium channels, which could explain the higher cardiotoxicity of cocaine–alcohol use compared to the use of cocaine alone. Insufficient data are available to outline the risk of cocaine–alcohol for cardiac arrhythmia.

### 3.6. Cocaine-Related ED Presentations and the Role of Alcohol Co-Use

As outlined above, cocaine increases myocardial oxygen deficiency, which is further enhanced when cocaine is combined with alcohol and may induce AMI, the most frequent severe cardiovascular complication in cocaine users [18,27], with a 4.3 to 24-fold increased risk of AMI within the first hour after cocaine use [17,18,19]. In patients hospitalized with cocaine-related AMI, ventricular arrhythmia occurred in 4–17% and congestive heart failure in 5–7%, while a fatal outcome of such complications was less than 2% (Hollander et al. 1995 [14]).

Table 1 lists the main findings from ED reports on the severity of complaints and mortality following cocaine–alcohol and cocaine alone. Based on urine analysis of cocaine metabolites, ED patients with acute drug overdose were stratified into cocaine alone (*n* = 150) and cocaine–alcohol (*n* = 49; assessed by CE in urine). CE-positivity was significantly associated with higher rates of cardiac arrest (6.1% vs. 0.7%, *p* = 0.048) and hyperlactatemia (4.1 mM vs. 2.9 mM, *p* = 0.038), but less myocardial injury (mean initial troponin 0.01 ng/mL vs. 0.16 ng/mL, *p* = 0.02) was observed [70]. Furthermore, among 245 homeless drug users, serum levels of CE were associated with serum troponin levels, a sensitive marker of myocardial ischemia (adjusted effect: 1.12; 95% CI:1.02–1.22) [71]. In 417 ED trauma patients, CE-positivity was associated with a more than five times higher risk of requiring intensive care assistance (OR = 5.9; 95% CI: 1.6–22) [72]. This study has, however, several noticeable limitations (see the section “Limitations of the Study”).

In another ED study assessing the clinical outcomes of 103 trauma patients and 212 non-trauma patients, cocaine–alcohol users in the *non-trauma group* were more likely to be admitted to an ICU than cocaine-alone users (37% vs. 7%; *p* < 0.05), but no significant differences were observed regarding chest pain [73]. In the *trauma group*, neither a significant difference in admission to an ICU nor a difference in the rate of chest pain was observed between cocaine–alcohol users and cocaine-alone users [73]. A recent Spanish ED study (*n* = 3925) failed to show higher cardiotoxicity for cocaine–alcohol: compared to cocaine alone, cocaine–alcohol did not increase the risk for chest pain (OR = 0.96; 95% CI: 0.64–1.45) [23]. Blaho et al. also observed among 111 ED patients with a cocaine-related intoxication no statistical correlations between the serum levels of cocaine, the cocaine metabolites, including CE, and the severity of the clinical symptoms, admission to ICU, the need for treatment, or the outcome [74]. Similarly, the severity of the clinical symptoms in 81 patients attending an ED in Brazil with cocaine intoxication was not related to the blood concentration of cocaine and/or CE [75]. This is consistent with the findings of Signs et al. [76] who reported no significant differences between cocaine–alcohol and cocaine alone with respect to cardiac and neurological complaints [76].

In summary, studies in patients attending an ED for cocaine-related medical complaints showed inconsistent findings with respect to whether a higher severity of symptoms was observed when alcohol was simultaneously used with cocaine: both increased and non-increased risks for cardiac complications and cardiac arrest were reported.

### 3.7. Cocaine-Related Mortality and the Role of Alcohol Co-Use

Cocaine-related death is most frequently caused by cardiac failure [8]. Following cocaine–heroin, or “speedballs”, cocaine–alcohol was the second most common two-drug combination in patients who died of substance abuse in the DAWN registry [77]. Moreover, cocaine–alcohol appeared to be associated with higher death rates than either agent alone (cf. Table 2).

The autopsy results of 49 cocaine-related postmortem cases of sudden and unexpected deaths between 2000 and 2011 in Australia showed that CE and ethanol were detected in 8 and 17 cases, respectively (51%) [78]. However, only 22 out of the 49 cases in this study were linked with drug toxicity. Ethanol was detected in 42% of cocaine-related deaths in a study in Texas, USA, between 1993 and 2005 (*n* = 461) [79], in 22% (*n* = 857) of cocaine-related deaths in England/Wales in 2022 [80], and in 35% (*n* = 72) of cocaine-related deaths in San Francisco [81]. These postmortem studies indicate the co-use of alcohol is often ascertained in cocaine-related death, which may have played a role in the cause of death.

Another ED study on 233 cocaine-using patients showed, on autopsy of fatal cases, that the cocaine blood level was lower in patients who used cocaine–alcohol compared with cocaine alone (0.9 versus 2.8 mg/L; *p* = 0.06) [13]. This large difference suggests a higher lethality of cocaine when used in combination with alcohol. A prospective case–control forensic autopsies study of cocaine-related sudden deaths (*n* = 21) between 2003 and 2006 in Spain showed the simultaneous use of alcohol in 16 cases. The main cause of sudden deaths was cardiovascular (13 cases) with left ventricular hypertrophy observed in 12 cases, small vessels disease in 9 cases, severe atherosclerotic coronary artery disease in 6 cases, and coronary thrombosis in 3 cases [82]. These findings suggest that myocardial ischemia may be the cause of cardiac arrest in these cocaine users, though the impact of smoking (81% of victims smoked tobacco) and comorbidity may have played a role as well (see study Limitations of the Study).

People with coronary artery disease, who had used cocaine–alcohol, had a 21.5 times greater risk for sudden death than those who had used cocaine alone [77], while in another study, CE was associated with a 25-fold increased risk of sudden cardiac death compared to cocaine alone [46]. An in-hospital mortality study performed in a national inpatient sample in the U.S. (*n* = 261,000,000; between 2006 and 2018) compared cocaine to cocaine–alcohol positive patients, admitted with a diagnosis of cocaine abuse, dependence, poisoning, or unspecified cocaine use. The results showed a higher rate of in-hospital mortality for cocaine–alcohol compared with cocaine alone (1.36% and 1.07%, respectively; *p* < 0.001), but this difference was no longer statistically significant (*p* = 0.87) after adjustment for a number of covariates, like age, sex, race, and comorbid conditions [9].

In summary, the combined use of cocaine and alcohol leads consistently to a higher mortality rate than the use of cocaine alone. The cause of death and the role of confounders, in general, is poorly or not reported.

## 4. Discussion

One of the most frequent drug use combinations leading to ED attendance is combined cocaine and alcohol use [20]. It is questioned whether the simultaneous use of cocaine and alcohol represents an additional cardiovascular risk compared to the use of cocaine alone, in particular, with respect to AMI and cardiac arrhythmia. Alcohol itself shows little direct cardiovascular effects [30], which rules out an additive cardiotoxic effect of alcohol when combined with cocaine. Moreover, various studies have observed a small protective acute effect of alcohol on cardiovascular events, including AMI and cardiac arrhythmia [67,83,84,85].

The current systematic review clearly shows a pharmacokinetic interaction between alcohol and cocaine, including the formation of CE, the typical metabolite formed by the simultaneous use of cocaine and alcohol. Simultaneous alcohol use results in a modest increase in cocaine plasma levels but does not largely change the cardiovascular effects induced by cocaine [46]. Compared to cocaine alone, cocaine–alcohol increased the HR and BP beyond the effects of both drugs individually, which may increase the risk of developing adverse cardiac events by increasing myocardial oxygen deficiency.

Inconsistent data on the risk of cardiotoxicity due to CE compared to cocaine alone were demonstrated. Some studies reported the cardiovascular effects of CE to be comparable, whereas other studies showed these effects to be smaller than those observed with cocaine alone [54,55,56,57,58,59], suggesting no higher cardiotoxicity due to cocaine–alcohol compared to cocaine alone. Additionally, the severity of clinical symptoms was not related to blood levels of either cocaine or CE [74,75,76]. Also, between cocaine alone and cocaine–alcohol, no significant difference was observed in their risk for developing chest pain [23,73]. Confusingly, on the one hand, CE was reported with both a lower [70] and a higher association with myocardial injury [71]. On the other hand, CE was reported to induce higher cardiotoxicity [53], higher rates of cardiac arrest and hyperlactatemia [70], higher admission to the ICU [72,73], and higher mortality in other studies [46,77].

The inconsistency in the findings with regard to the cardiac risk of cocaine–alcohol may also be driven by heterogeneity in the patient characteristics (e.g., age), risk behavior (e.g., chronic smoking, chronic alcohol, or polydrug use), and traditional cardiovascular risk factors (e.g., morbidities) [6]. Polydrug use is known to be highly prevalent among cocaine users [2,86] and it is well-known that the simultaneous use of cocaine and other substances may exacerbate cocaine-associated cardiovascular complications [87]. In addition, prolonged cocaine use may predispose to cardiovascular disease, like coronary and myocardium abnormalities [87,88,89].

Epidemiologic studies strongly support the assertion that cigarette smoking increases the incidence of myocardial infarction and fatal coronary artery disease [90]. This is important because a substantial part of cocaine users consists of regular smokers [17,91]. Chronic smoking exacerbates the deleterious effects of cocaine on myocardial oxygen supply and demand [10,92] since the combination results in greater increases in HR and coronary vasoconstriction than either alone [93].

It is concluded that cocaine–alcohol is associated with an increased mortality risk compared to using cocaine alone, although the underlying mechanism is not well understood. The high rate of sudden death following cocaine–alcohol is probably caused by cardiac complications, like cardiac arrest (e.g., [82]). To ascertain this increased risk, it is advocated to perform more appropriate and accurate registrations, including drug use, smoking behavior, and pre-existing physical comorbidities for patients presenting to the ED with chest pain after cocaine or other substance use. In addition to standard blood analyses for cocaine, its metabolites and CE [14,94], patients should be questioned about their alcohol use, recreational drug use, and smoking history.

## 5. Limitations of the Study

Unfortunately, the reports on cardiovascular complications in cocaine users and cocaine–alcohol co-users in the ED showed highly inconsistent findings and large variability, probably due to poor stratification of cocaine users with respect to a variety of potential confounders (e.g., [9,82]. The low quality of the data frustrates a sound answer to the question of whether cocaine–alcohol poses a larger cardiovascular risk than the use of cocaine alone. Numerous studies showed that chronic cocaine use was associated with chronic cardiovascular conditions such as cardiomyopathy, subclinical atherosclerosis, and coronary artery disease (e.g., Kim and Park 2019 [4]), implying that the frequency of cocaine use could be an important factor affecting clinical complaints and mortality risk among cocaine users.

Furthermore, wide variability exists in patients presenting at the ED with cocaine-associated chest pain, including differences in the amounts used, the route of administration, the time since last use, a history of chronic use, premorbid cardiac disease and cardiac risk factors, and individual differences in cocaine metabolism [95]. Also, differences in the route of administration may affect the outcome because the oral route results in significantly greater cocaethylene formation than smoking (34% versus 18%) [44]. Urine drug testing is not always available in the ED, so the practitioner relies on the patient’s history, which is often inaccurate. Moreover, in some studies, CE has been found to have low validity considering its poor or inconsistent correlation with the level of cocaine (r = 0.02), even when subjects were stratified by alcohol level [96]. However, others have found better correlations between CE and cocaine plasma levels (r = 0.42; *p* < 0.01) and urine levels (r = 0.83, *p* < 0.01) [97].

In general, the quality of mortality studies and ED studies is quite poor because data about confounders like a history of cocaine use, a smoking history, polydrug use, and traditional cardiovascular risk factors were rarely reported. The study by Wiener et al. [72] on ED patients with trauma is another example of a confounded approach, considering that the trauma may have directly resulted from violent incidents caused by the combined use of alcohol and cocaine, i.e., not by adverse cardiovascular events. As rightly concluded by Signs et al. [76], ED patients who have concurrently consumed cocaine and alcohol present to the ED more often with complaints related to traumatic injury compared to those related to toxicologic complications.

Based on the data presented, we conclude that the simultaneous use of alcohol by cocaine users is a serious risk factor for acute cardiac death. ED patients with chest pain after alcohol and cocaine co-use, that are stratified as low risk, should be considered for prolonged monitoring due to the prolonged risk of serious cardiovascular complications due to pharmacokinetic and pharmacodynamic interactions.

## Figures and Tables

**Table 1:** Main findings from ED reports and mortality data related to the use of cocaine alone and co-use of alcohol and cocaine (cocaine–alcohol).

Sample Characteristics	Sample Size	Main Findings	Reference
ED patients	199	CE-positive serum (cocaine–alcohol) compared with CE-negative serum (cocaine alone): higher rates of cardiac arrest (6.1% vs. 0.7%, *p* = 0.048) and hyperlactatemia (4.1 mM vs. 2.9 mM; *p* = 0.038) but lower rate of myocardial injury (mean initial troponin 0.01 ng/mL vs. 0.16 ng/mL; *p* = 0.02)	[70]
Homeless drug users	245	Serum levels of CE were associated with serum troponin levels, a sensitive marker of myocardial ischemia (adjusted effect: 1.12; 95% CI:1.02–1.22)	[71]
ED patients	417	CE-positivity associated with risk of requiring ICU assistance (OR = 5.9; 95% CI: 1.6–22)	[72]
ED patients	212	Drug screen: Cocaine–alcohol vs. cocaine alone: more often confusion (28% vs. 9.5%), higher ICU admission (48% vs. 31%), no significant difference in the rate of chest pain	[73]
ED patients	3925	No difference in chest pain between cocaine–alcohol use and cocaine-alone use (OR = 0.96; 95% CI: 0.64–1.45)	[23]
ED patients	111	No relation between serum levels of CE and cocaine metabolites and severity of intoxication, clinical symptoms, admission to ICU, or need for treatment	[74]
ED patients	81	No relation between the blood levels of cocaine CE with severity of stimulant intoxication	[75]
ED patients	228	No significant differences in cardiac and neurological complaints between cocaine and cocaine–alcohol intoxication	[76]

CE: cocaethylene; ICU: intensive care unit.

**Table 2:** Mortality in subjects who had used cocaine in combination with alcohol.

Sample Characteristics	Sample Size	Main Findings	Reference
Postmortem evidence	49	Cases of sudden and unexpected death between 2000 and 2011 in Australia: cocaine–alcohol in 17 of 49 cases (35%)	[78]
Postmortem evidence	461	Ethanol was detected in 194 of 461 cocaine-related deaths (42%)	[79]
Postmortem evidence	857	Ethanol was detected in 189 of 857 cocaine-related deaths (22%) in England/Wales in 2022	[80]
Postmortem evidence	72	Ethanol was detected in 25 of 72 cocaine-related deaths (35%)	[81]
Postmortem evidence	Not reported	Lower cocaine blood level in patients who had also used alcohol (0.9 versus 2.8 mg/L; *p* = 0.06)	[13]
Postmortem evidence	Not reported	Cocaine–alcohol increased the risk of sudden death 18-fold	[77]
Postmortem evidence	21	Cocaine-related sudden deaths: cocaine–alcohol in 76% and cigarette smoking in 81% of cases	[82]
Postmortem evidence	Not reported	Cocaethylene was associated with an 18 to 25-fold higher risk of acute cardiac death compared to cases using cocaine alone	[46]
In-hospital mortality	2,368,886	Non-significantly lower in-hospital mortality rate for cocaine–alcohol than for cocaine alone: 1.07% vs. 1.34% (aOR = 0.99; *p* = 0.87)	[9]

## Data Availability

Not applicable.

## Supplementary Materials

The following supporting information can be downloaded at: https://www.mdpi.com/article/10.3390/jcm13051475/s1, Search string and PRISMA checklist.
